# Chromosomal Signatures Corroborate the Phylogenetic Relationships within Akodontini (Rodentia, Sigmodontinae)

**DOI:** 10.3390/ijms21072415

**Published:** 2020-03-31

**Authors:** Willam Oliveira da Silva, Stella Miranda Malcher, Adenilson Leão Pereira, Julio Cesar Pieczarka, Malcolm Andrew Ferguson-Smith, Patricia Caroline Mary O’Brien, Ana Cristina Mendes-Oliveira, Lena Geise, Cleusa Yoshiko Nagamachi

**Affiliations:** 1 Laboratório de Citogenética, Centro de Estudos Avançados da Biodiversidade, Instituto de Ciências Biológicas, Universidade Federal do Pará (UFPA), Belém, Pará, 66075-750, Brazil; willam_oliveira@hotmail.com (W.O.d.S.); stella.malcher@hotmail.com (S.M.M.); adenilson.leao@hotmail.com (A.L.P.); juliopieczarka@gmail.com (J.C.P.); 2 Cambridge Resource Centre for Comparative Genomics, Department of Veterinary Medicine, University of Cambridge, Cambridge 01223, UK; maf12@cam.ac.uk (M.A.F.-S.); allsorter@gmail.com (P.C.M.O.); 3 Laboratório de Zoologia e Ecologia de Vertebrados, ICB, Universidade Federal do Pará (UFPA), Belém, Pará, 66075-110, Brazil; cris.mastozoologia@gmail.com; 4 Departamento de Zoologia, Laboratório de Mastozoologia, Universidade do Estado do Rio de Janeiro, Rio de Janeiro, 20550-170, Brazil; lenageise@gmail.com

**Keywords:** conserved syntenic block, chromosome painting, rodents, FISH

## Abstract

Comparative chromosome-painting analysis among highly rearranged karyotypes of Sigmodontinae rodents (Rodentia, Cricetidae) detects conserved syntenic blocks, which are proposed as chromosomal signatures and can be used as phylogenetic markers. In the Akodontini tribe, the molecular topology (Cytb and/or IRBP) shows five low-supported clades (divisions: “*Akodon*”, “*Bibimys*”, “*Blarinomys*”, “*Oxymycterus*”, and “*Scapteromys*”) within two high-supported major clades (clade A: “*Akodon*”, “*Bibimys*”, and “*Oxymycterus*”; clade B: “*Blarinomys*” and “*Scapteromys*”). Here, we examine the chromosomal signatures of the Akodontini tribe by using *Hylaeamys* *megacephalus* (HME) probes to study the karyotypes of *Oxymycterus amazonicus* (2n = 54, FN = 64) and *Blarinomys breviceps* (2n = 28, FN = 50), and compare these data with those from other taxa investigated using the same set of probes. We strategically employ the chromosomal signatures to elucidate phylogenetic relationships among the Akodontini. When we follow the evolution of chromosomal signature states, we find that the cytogenetic data corroborate the current molecular relationships in clade A nodes. We discuss the distinct events that caused karyotypic variability in the *Oxymycterus* and *Blarinomys* genera. In addition, we propose that *Blarinomys* may constitute a species complex, and that the taxonomy should be revised to better delimit the geographical boundaries and their taxonomic status.

## 1. Introduction

Chromosome painting (Fluorescence *In Situ* Hybridization–FISH-with whole-chromosome probes) can be used to investigate genomic changes among different groups of vertebrates. This approach has improved our understanding of the evolutionary processes ranging from speciation [1] to ancestral karyotype hypotheses [2,3]; it allows the comparison of homeologies between species with evolutionary distances of about 55 Ma (million years ago). Comparative chromosome-painting analysis also identifies chromosome segments that have preserved synteny throughout evolution [4], without the need for complex mapping for each species [5], even allowing the determination of putative ancestral karyotypes [6]. For instance, in Xenarthra (sloths, armadillos, and anteaters), the hybridization of human whole-chromosome probes (HSA) in *Bradypus torquatus* and *B. variegatus* revealed that the syntenic association of HSA 17/19 is an exclusive trait of the *Bradypus* genus, leading to the proposal of a hypothetical Xenarthran karyotype with 48 chromosomes distinct from the ancestral Eutherian karyotype. Moreover, trait HSA 7/10 and the fragmentation of HSA 8 into three blocks were suggested to support the monophyly of Xenarthra [3].

In some mammalian orders that exhibit extensive karyotypic diversification (e.g., Artiodactyla, Carnivora, Chiroptera, and Rodentia), comparative analysis of chromosome-painting data has revealed the occurrence of complex rearrangements that were not identified by classical cytogenetics alone (conventional staining, C-, G-banding) [7,8,9,10,11,12,13]. The Asian muntjacs (Artiodactyla) exhibit chromosomal variability from 2n = 6/7 in *Muntiacus muntjak vaginalis* to 2n = 46 in *Muntiacus reevesi*; this was most likely caused by tandem and centromeric fusions from a hypothetical ancestral karyotype with 46 chromosomes [12]. Within Carnivora, Canidae exhibit more than 40 chromosomal changes from the ancestral carnivore karyotype, with several intrachromosomal rearrangements revealed by Bacterial Artificial Chromosome (BAC) mapping [13].

In general, chromosomal rearrangements are rare events with low levels of homoplasy [14]. Despite this, in some vertebrate lineages, chromosomal number and morphology are highly variable [2,12,13,15]. Several studies using chromosome painting were able to detect conserved syntenic associations shared among distinct taxa (chromosomal signatures). These signatures have been used in the construction of chromosomal topologies and as phylogenetic markers, as they are often group-specific, evolutionarily conserved, and phylogenetically informative, and corroborate the phylogenetic relationships obtained from molecular topologies [2,3,9,10,12,13,14,15,16,17]. As an example, the monophyly of Afrotheria was debated due to the lack of morphological evidence, but the identification of syntenic associations HSA 1/19 and 5/21 provided support for the Afrotheria monophyly [5]. Understanding how these rearrangements occur and what they mean in evolutionary terms can be answered in the near future by a chromosomics approach, in which sequencing and epigenetics can be used to comprehend why some rearrangements are conserved while others are not [18].

In rodents, chromosome-painting investigations have been carried out in more than 100 species [15], a relatively small proportion of the more than 2000 species in this group [19,20]. Data obtained with whole-chromosome probes from *Mus musculus* (MMU) were used to propose chromosomal signatures MMU 3/18 and 6/12 for the Sigmodontinae subfamily (Rodentia, Cricetidae) [7,20,21] but, considering that the mouse chromosomes are highly reorganized, the authors did not attempt to reconstruct the putative ancestral karyotype of this subfamily. More recently, Sigmodontinae rodents have been analyzed by chromosome painting with whole-chromosome probes of a subfamily member, *Hylaeamys megacephalus* (HME) on representatives of the Oryzomyini and Akodontini lineages [8,9,22,23,24,25,26]. These studies have shed light on the karyotype evolution of New World rodents demonstrating syntenic associations for Sigmodontinae: HME 7/(9,10), 1/12, 6/21, 20/(13,22), 19/14/19, 8, 11/(16,17), 5/(16,17), 15, 24, and 26. In Akodontini, four species of three genera (*Akodon*, *Thaptomys*, and *Necromys*) were analyzed and the associations HME 2/18, 3/25, 18/25, and 4/11/(16,17) were identified as traits for this tribe [25].

The Akodontini are the second most diverse tribe of the Sigmodontinae (Rodentia, Cricetidae); 85 species have been organized into 15 genera, all of which occur in South America [20]. Although no published phylogeny has included all members and the relationships among the taxa remain unclear [20], five clades have been proposed (referred to as “divisions”) [27]: “*Akodon*”, “*Bibimys*”, “*Blarinomys*”, “*Oxymycterus*”, and “*Scapteromys*” (Table 1). The phylogenetic relationships among some genera exhibited low-supported nodes, but two high-supported major clades were recovered, one formed by the *Akodon*, *Bibimys*, and *Oxymycterus* divisions (clade A), and the other formed by the *Blarinomys* and *Scapteromys* divisions (clade B).

In the Akodontini, the diploid number (2n) ranges from 2n = 10 in *Akodon* sp. to 2n = 54 in *Oxymycterus* sp. [29,30]. It has been proposed that the reduction in 2n is the chromosomal evolutionary trend of this tribe: the putative ancestral karyotype has a high 2n (58) with numerous one-armed chromosomes, whereas the derived karyotypes generally have more bi-armed chromosomes arising from Robertsonian translocations [31,32,33,34].

Here, we focused on two Akodontini genera that present distinct chromosomal evolutionary patterns: *Oxymycterus* and *Blarinomys*. The genus *Oxymycterus* has 16 valid species [20,35]. Cytogenetic data are available for seven of them, which have a consistent 2n (54) but fundamental autosomal number (FN) ranging from 60 to 64 (Table 2). The cytogenetic studies in *Oxymycterus* have been limited to classical cytogenetics (conventional staining and chromosomal banding); in most cases, the 2n and FN were reported without showing the karyotype.

The *Blarinomys* genus is considered monotypic (*B. breviceps*), with two main well-structured clades identified by phylogenetic reconstructions (Maximum Likelihood, Maximum Parsimony, and Bayesian Inference analysis of Cytb gene); it has eight chromosomal forms with 2n ranging from 28 to 52, a consistent FN of 50, and a varied number of B chromosomes (from 0 to 4). *Blarinomys breviceps* karyomorphs were analyzed by C-banding, G-banding, R-banding, and FISH with telomeric probes that showed the presence of interstitial telomeric sequences (ITS), but these methods were not employed in all specimens. For example, the karyotype with 2n = 28, FN = 50 was assessed only by G-banding and telomeric FISH techniques [42].

Taking into account that comparative cytogenetic data are often phylogenetically informative [14] and chromosomal signatures are maintained in rodent lineages regardless of the high rate of chromosomal change that may occur within each group [9,25], we set out to detect chromosomal signatures that can be used as phylogenetic markers to elucidate the phylogenetic relationships of some Akodontini members. Towards this, we performed comparative chromosome painting using HME whole-chromosome probes [23] on representatives from three Akodontini divisions [27]: *Oxymycterus amazonicus* (*Oxymycterus* division; present work), *Blarinomys breviceps* (*Blarinomys* division; present work), *Akodon* sp., *A. montensis*, *Necromys lasiurus*, and *Thaptomys nigrita* (*Akodon* division) [24,25]; we also compared these species with data obtained using HME probes in other taxa [8,9,23,26].

**Figure 1:**
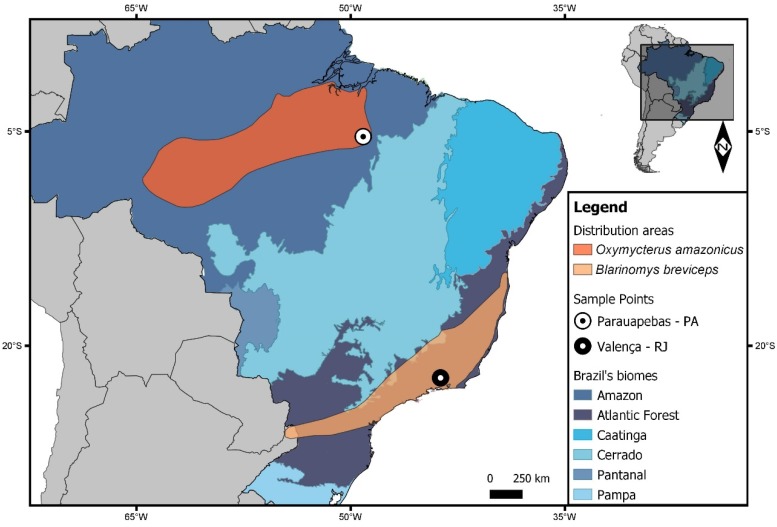
Map showing the distribution areas and sampling points for *Oxymycterus amazonicus* and *Blarinomys breviceps*. The Brazilian states are Pará (PA) and Rio de Janeiro (RJ). Biomes from Brazil are shown in different colors. Other South American countries are shown in gray. The map was made using QUANTUM-GIS (Q-GIS) v. 3.8.0. The database was obtained from DIVA and REDLIST.

Here, we describe new cytogenetic results for *Oxymycterus* and *Blarinomys* collected in distinct localities of Brazil (Figure 1), and use the chromosomal signatures detected to elucidate the phylogenetic relationships within Akodontini clade A.

## 2. Results

It should be noted that the heterochromatic regions do not exhibit hybridization signals by FISH analysis with *Hylaeamys megacephalus* (HME) whole-chromosome probes.

### 2.1. Oxymycterus Amazonicus (OAM; 2n = 54, FN = 64)

**Figure 2:**
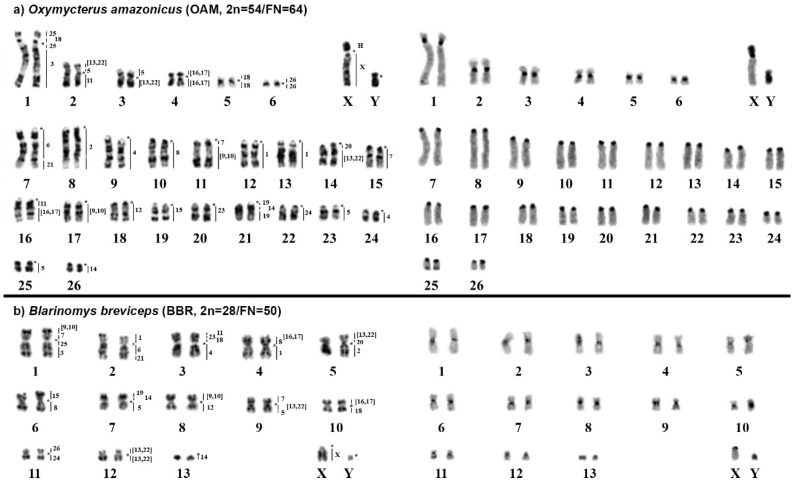
Karyotypes of (**a**) *Oxymycterus amazonicus* and (**b**) *Blarinomys breviceps*. The G-banded karyotypes with chromosome painting performed using *Hylaeamys megacephalus* probes [23] are shown in the left panel, while the C-banded karyotypes are shown in the right panel. An asterisk indicates a centromere, while “H” indicates a large block of constitutive heterochromatin.

The karyotype of *Oxymycterus amazonicus* (OAM) has 2n = 54 and FN = 64 with autosomes comprising six meta/submetacentric pairs (pairs 1 to 6) and 20 acrocentric pairs (pairs 7 to 26); the X chromosome is a large submetacentric, and the Y chromosome is a small submetacentric. The constitutive heterochromatin is distributed at the centromeric region of all autosomes. The X chromosome carries a large heterochromatic block in the short arm, while the Y chromosome is almost entirely heterochromatic (Figure 2a).

**Figure 3:**
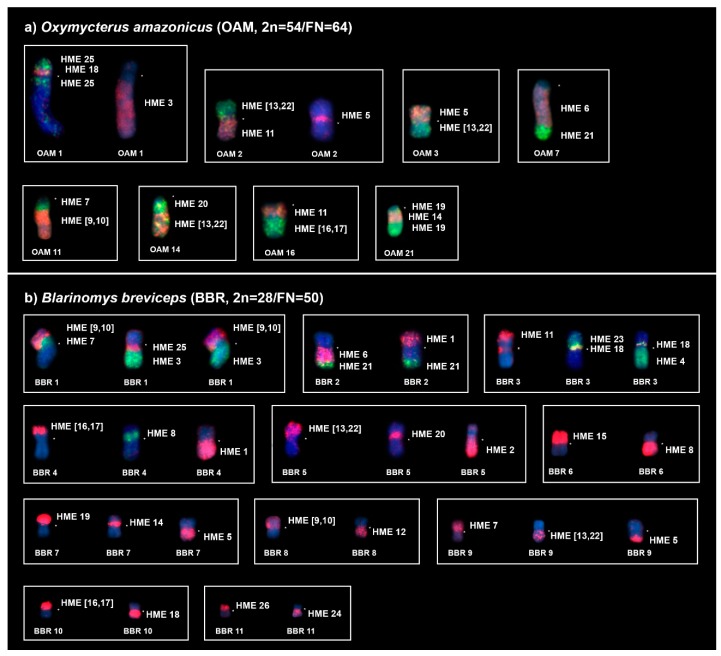
Chromosomal associations obtained from (**a**) *Oxymycterus amazonicus* (OAM) and (**b**) *Blarinomys breviceps* (BBR) using HME probes [23]. Each box corresponds to a chromosome pair that is shown in Figure 2 and exhibits chromosomal associations; for some chromosome pairs, single or multiple chromosomes are shown with different probes to exhibit that the HME whole-chromosome probes covered the entire chromosome. An asterisk indicates a centromere. HME whole-chromosome probes are shown as red (CY3), green (FITC), and yellow (CY3 + FITC); the counterstaining is blue (DAPI).

FISH with HME probes showed 39 hybridization signals in OAM (Figure 2a, Table 3). Twelve autosomes showed conserved synteny; of them, seven (HME 2, 8, 12, 15, 23, 24, and 26) hybridized to whole chromosomes of OAM (8, 10, 18, 19, 20, 22, and 6, respectively), and five (HME 3, 6, 19, 20, 21) hybridized to parts of other chromosomes (OAM 1q distal, 7q proximal, 21q proximal and distal, 14q proximal and 7q distal, respectively). The other 11 autosomal probes showed multiple signals in OAM; nine (HME 1, 4, 7, (9,10), 11, 14, (16,17), 18, and 25) hybridized to two chromosomes each, while HME (13, 22) showed signals in three chromosomes and HME 5 showed signals in four chromosomes. The X chromosome hybridized to OAM Xq due to the presence of a large heterochromatic block at OAM Xp. Eight OAM pairs showed chromosomal (syntenic) associations (Figure 2a and Figure 3a).

### 2.2. Blarinomys Breviceps (BBR; 2n = 28, FN = 50)

The karyotype of *Blarinomys breviceps* (BBR) has 2n = 28 and FN = 50, with autosomes comprising 12 meta/submetacentric pairs (pairs 1 to 12) and one acrocentric pair (pair 13); the X chromosome is a middle-sized acrocentric, and the Y chromosome is a small acrocentric. The constitutive heterochromatin is distributed at the centromeric region of all autosomes and the X chromosome; the Y chromosome is almost entirely heterochromatic (Figure 2b).

FISH with HME probes showed 34 hybridization signals in BBR (Figure 2b, Table 3). Fifteen probes showed conserved synteny (14 autosomal probes plus the X chromosome); all 14 autosomal probes (HME 2, 3, 4, 6, 11, 12, 15, 19, 20, 21, 23, 24, 25, and 26) hybridized to parts of other chromosomes (BBR 5p, 1q distal, 3q, 2q proximal, 3p distal, 8q, 6p, 7p distal, 5p proximal, 2q distal, 3p interstitial, 11q, 1q proximal, and 11p, respectively). The other nine autosomal probes showed more than one signal in BBR, with eight (HME 1, 5, 7, 8, (9,10), 14, (16,17), and 18) hybridizing to two chromosomes each, while HME (13,22) hybridized to three chromosomes. Eleven BBR pairs showed chromosomal associations (Figure 2b and Figure 3b).

## 3. Discussion

### 3.1. New Cytogenetic Data for Oxymycterus and Blarinomys

In the last two decades, the genus *Oxymycterus* has undergone several changes in taxonomy, with increases in the number of species and many synonyms for its representatives [20]. Although the 16 valid species of *Oxymycterus* exhibit variability in diagnostic morphological features [20,35], some studies use obsolete names for some taxa that make it difficult to assign karyotypes to species, given that the group has very low cytogenetic variability (2n = 54, FN = 60-64). Here, we adopt the taxonomic classification proposed by Patton, Pardiñas, and D’Elía [20] (Table 2).

In attempting to review the cytogenetic data of *Oxymycterus*, Di-Nizo et al. [43] listed 2n = 54 for *O. amazonicus*; however, the sources cited for this information do not describe the karyotype of this taxon [19,36]. In fact, Bonvicino et al. [36] compared *O. quaestor* and *O. caparaoe* G-banded karyotypes (which were not shown) with the cytogenetic information of *O. dasythricus*, *O. delator*, *O. nasutus*, *O. paramensis*, and *O. rufus*, and proposed that the entire genus is composed of a single karyotype (2n = 54, FN = 64). Here, we describe for the first time the karyotype of *O. amazonicus* (2n = 54, FN = 64) assessed by classic banding and chromosome painting. Our study of the genus revealed that the available *Oxymycterus* karyotypes vary in FN from 60 to 64 (Table 2), exhibit at least one large submetacentric (pair 1), and range from three to five bi-armed chromosomes. This suggests that pericentric inversions, translocations, or centromeric repositioning could explain why the 2n remains consistent while the FN varies [29,31,38,39,40,44], which disagrees with the proposition of Bonvicino et al. [36]. 

In relation to sex chromosome morphology, we observed three types of X: 1) medium metacentric (*O. dasytrichus*), 2) large subtelocentric (*O. delator*), and 3) large submetacentric (*Oxymycterus* sp., *O. amazonicus*, *O. caparaoe*, *O. paramensis*, *O. quaestor*, and *O. rufus*). C-banding data for *O. amazonicus* (present study) and *Oxymycterus* sp. [29] show the presence of a large heterochromatic block in the short arm; this suggests that amplification/deletion of constitutive heterochromatin probably accounts for the difference between subtelocentric and submetacentric morphologies, while the medium metacentric is probably due to pericentric inversion or centromeric repositioning [44]. The Y chromosome is described as acrocentric for five species (*O. caparaoe*, *O. dasytrichus*, *O. paramensis*, *O. quaestor*, and *O. rufus*), while the Y-chromosomes of *Oxymycterus* sp. and *O*. *amazonicus* have a submetacentric morphology that is probably due to pericentric inversion or centromeric repositioning [44].

Among the eight karyomorphs of *Blarinomys breviceps* (2n = 28-52, FN = 50), more one-armed chromosomes are seen in those with a higher 2n, whereas more bi-armed chromosomes are seen in karyotypes with a lower 2n [42]. Ventura et al. [42] recovered two major clades in a phylogenetic reconstruction of *B. breviceps* specimens: the northeastern lineage comprised samples with 2n = 52, while the southeastern lineage exhibited a higher diversity of 2n (ranging from 28 to 45). Although the authors did not speculate on the direction of chromosomal change in the southeastern lineage, we believe that this group experienced several Robertsonian translocations that caused the lower 2n. This is consistent with a previous proposition regarding the evolutionary trend of Akodontini members [34], as seen in *Akodon* [21].

We also compared the distribution of constitutive heterochromatin and noticed that four previously reported karyomorphs (2n = 52, 43, 37, 31) had heterochromatin signals in the pericentromeric regions of only some autosomes [42], while our karyotype (2n = 28; MN68882) presented heterochromatin in all autosomes. Differences in the amount of constitutive heterochromatin in karyotypes of the same species or genus are frequent in rodents [45], as observed in *Neacomys* that showed heterochromatin blocks in from three to five bi-armed pairs among samples from distinct localities in eastern Amazon [9,46]. 

In *Blarinomys*, the genetic divergence ranges from 4.8% to 8.4% [42], and several morphological traits vary between the northeastern and southeastern clades [20]. This level of genetic divergence is consistent with the range established for potentially undescribed species (over 5%) [47,48]. This suggests that *Blarinomys* constitutes a species complex, and that the taxonomy should be revised to take into account geographical boundaries.

### 3.2. Speciation Hypothesis in Oxymycterus and Blarinomys

*Oxymycterus* presents distinct morphological features and molecular variation among its representatives [20], with low chromosomal variability (2n = 54, FN = 60–64; Table 2). It also exhibits great adaptability, being widely distributed in distinct environments of South America (Amazon Forest, Atlantic Forest, Caatinga, Cerrado, Pampas, Chaco, and Andes), and at elevations ranging from sea level to about 3500 m in the montane forests [20]. Some species have isolated distributions, while others may occur in sympatry with two or even six species [20]. 

The literature does not contain any phylogeographic study that covers all representatives of the *Oxymycterus* genus. Peçanha et al. [49] investigated the phylogeographic history of *Oxymycterus nasutus*, which occurs in the Atlantic Forest and Pampas biomes. The authors recovered six major structured populations that exhibited morphological and molecular differences. They proposed that there had been a population expansion with late retraction during the postglacial period, and concluded that the observed structure was a result of vicariance events with refuge isolation.

Similarly, we hypothesized that the speciation process of *Oxymycterus* was developed by vicariance, occurring through ecological adaptations to the distinct and complex biogeographic dynamics that occurred in South America [50]. Ecological studies in *Oxymycterus* demonstrated that the species of this genus are not affected by habitat fragmentation [51]; this could explain the chromosomal stability of this genus, since its members tend to be organized in larger populations. This suggests that chromosomal rearrangements have not played a crucial role in the speciation process of *Oxymycterus*. However, we emphasize that only after detailed phylogeographic studies of the *Oxymycterus* genus will it be possible to fully comprehend the speciation process of this understudied group [52].

The *Blarinomys* genus occurs in the Atlantic Forest biome (Figure 1) and is separated into two lineages: the northeastern and southeastern clades [42]. This separation is in accordance with two phylogeographic regions that are recognized in the Atlantic Forest [53] and are attributed to the presence of the Rio Doce [54]. Similar separations have been described for other groups of terrestrial vertebrates (e.g., amphibians, birds, and small mammals) in this region [55,56,57].

Although the Atlantic Forest is considered a hotspot for biodiversity [58], it is one of the most anthropized biomes in the world [59]. Due to five centuries of human expansion and degradation [60], this biome is now represented by small and isolated fragments that comprise only 11.7 % of its original cover [61].

The literature lacks any population dynamic data for the *Blarinomys* genus. Haag et al. [62] used microsatellite and mitochondrial markers to investigate the impact of Atlantic Forest fragmentation on *Panthera onca* samples. Although *P. onca* has a high dispersal capability, Haag et al. [62] identified decreases in population size and genetic diversity and probable impediments in gene flow. Thus, there appears to be limited dispersion across separate fragments that contain small and isolated populations, which are suffering from the effects of genetic drift. The impact of this fragmentation has been observed in large carnivores and is likely to be much more intense in rodents. As discussed before [11], some biological features of rodents allow the isolation of populations, since many generations can be produced in a short period of time, due to an elevated reproductive rate and a short pregnancy with the birth of large numbers of individuals per gestation. In addition, the low vagility of rodents favors endogamy [63]. This increases the probability of interbreeding between individuals heterozygous for a rearranged chromosomal form, which could lead to the development of homozygous subpopulations within a few generations [64,65].

*Blarinomys* exhibits high levels of morphological and molecular divergence between, but not within, the northeastern and southeastern clades [42], indicating that the Rio Doce has acted as an allopatric barrier to gene flow between these two lineages [54]. Carnaval et al. [55] demonstrated that the portion of the forest northeastern of the Rio Doce is highly stable, while the portion to the southeast is unstable. The northeastern populations exhibit karyotypic stability, which is compatible with a stable environment. However, the southeastern lineage exhibits a high level of chromosomal variability, with 2n ranging from 28 to 45. This indicates that chromosomal rearrangements played an important role in the separation of populations, and that the fragmentation (instability) of the Atlantic Forest reinforced the isolation of distinct populations with established karyotypes.

### 3.3. Chromosomal Signatures in Akodontini Reinforce Clade A Monophyly

Above, we discussed the chromosomal evolution of *Oxymycterus* and *Blarinomys* using only classical cytogenetics data to predict the events that may have occurred within both genera. Here, by analyzing OAM and BBR chromosome-painting data (Table 3) and comparing them with karyotypes available in the literature [19,21,33,38,39,40,42], we reaffirm that *Oxymycterus* and *Blarinomys* had distinct and independent chromosomal evolutionary processes: the former exhibits chromosomal stability with few inversions and/or translocations, while the latter is marked by numerous Robertsonian translocations that decrease the 2n in this group.

Chromosome-painting data and those obtained using HME whole-chromosome probes were compared between representatives of the Oryzomyini and Akodontini tribes (Supplementary Table S1). However, we will not discuss herein the signatures of Oryzomyini and its representatives, as we found nothing that we could add to the previous discussion of this from the results with HME probes [8,9,23,24,25,26]. Rather, we confirm their proposals about the ancestral traits for the Sigmodontinae (HME 7/(9,10), 8, 1/12, 6/21, 11/(16,17), 5/(16,17), 20/(13,22), 15, 19/14/19, 24, and 26) and Oryzomyini (HME 8a, 8b, 18, and 25).

**Figure 4:**
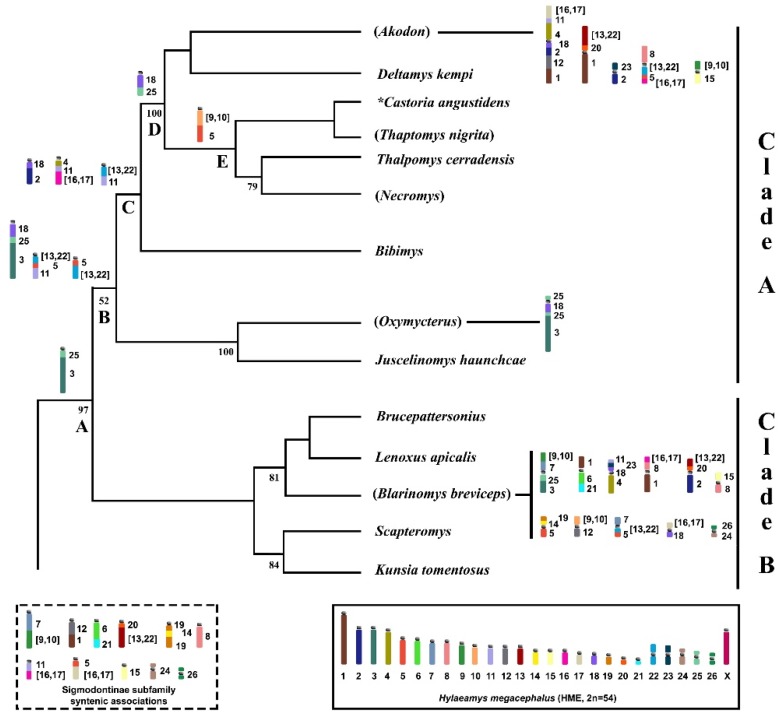
Part of the phylogeny from D’Elía [27] of the Akodontini tribe obtained by a Maximum Parsimony analysis (Cytb and/or IRBP) with modifications exhibiting sister clades A and B. Numbers below branches indicate parsimony jackknife values. Only values above 50% are shown. The box encloses an idiogram of the HME karyotype elaborated by Oliveira da Silva et al. [9], as assessed based on HME probes [23]. Idiograms above nodes indicate syntenic associations shared among lineages, which are based on taxa analyzed by chromosome painting with HME probes and G-banding patterns: Node A (Akodontini tribe), node B (clade A), node C (*Akodon*, *Deltamys*, *Castoria*, *Thaptomys*, *Thalpomys*, *Necromys*, and *Bibimys*), node D (*Akodon*, *Deltamys*, *Castoria*, *Thaptomys*, *Thalpomys*, and *Necromys*), node E (*Castoria*, *Thaptomys*, *Thalpomys*, and *Necromys*). Idiograms within dashed line correspond to syntenic associations for the Sigmodontinae subfamily. Idiograms beside taxa names indicate autapomorphic characters. Taxa analyzed by chromosome painting with HME probes are shown within parentheses. *Referred to as “*Akodon*” *serrensis* by D’Elía [27].

The Akodontini topology recovered by D’Elía [27] using a Maximum Parsimony strict consensus tree (Cytb and/or IRBP) exhibited five weakly supported divisions (*Akodon*, *Bibimys*, *Blarinomys*, *Oxymycterus*, and *Scapteromys*). The authors noted that further analysis would be needed to clarify the phylogenetic relationships among the genera, but that two major clades were recovered with a jackknife support value of 97%: clade A (*Akodon*, *Bibimys*, and *Oxymycterus* divisions) and clade B (*Blarinomys* and *Scapteromys* divisions) (Table 1, Figure 4).

In an attempt to understand the evolutionary process from the karyotypic changes observed in *A*. *montensis* (AMO), *Thaptomys nigrita* (TNI) [24], *Akodon* sp. (ASP), *Necromys lasiurus* (NLA) [25], *Oxymycterus amazonicus* (OAM), and *Blarinomys breviceps* (BBR) (present study), we detected chromosomal signatures that could clarify relationships within clade A. We focused on this clade because we had data from five species (ASP, AMO, TNI, NLA, OAM) from four (*Akodon*, *Thaptomys*, *Necromys*, and *Oxymycterus*) of the nine genera of clade A, but only one species (BBR) of five genera from clade B. Thus, any assumptions made regarding clade B would be only weakly supported.

The relationships amongst the genera of clade A are as follows: ((*Oxymycterus* + *Juscelinomys*) (*Bibimys*) (((*Castoria* + *Thaptomys*) (*Necromys* + *Thalpomys*)) (*Akodon* + *Deltamys*))) [27]. Only four nodes exhibited support values above 50%: the cluster (*Oxymycterus* + *Juscelinomys*) and the other separating (*Akodon* + *Deltamys*) from the clade ((*Necromys* + *Thalpomys*) (*Castoria* + *Thaptomys*)) are shown with 100% each; support value of 79% was present in the clade (*Necromys* + *Thalpomys*), while the first branch separating (*Oxymycterus* + *Juscelinomys*) from ((*Bibimys*) (((*Castoria* + *Thaptomys*) (*Necromys* + *Thalpomys*)) (*Akodon* + *Deltamys*))) exhibited 52% [27]. We put the chromosomal information into this topology and followed the direction of chromosomal change (discussed below) and noticed that the chromosomal signatures retrieved the same relationships as recovered by molecular data, with no major alterations (Figure 4): ((*Bibimys*) (*Oxymycterus* + *Juscelinomys*) (((*Necromys* + *Thalpomys*) (*Castoria* + *Thaptomys*)) (*Akodon* + *Deltamys*))). 

Only the trait HME 3/25 (Node A, Figure 4) is shared between clades A and B, which is a chromosomal signature for the Akodontini tribe [3,13]. The clade A node retrieved the chromosomal signatures HME 18/25/3, (13,22)/5/11, and 5/(13,22) (Node B, Figure 4); the following branch is composed of the *Oxymycterus* and *Juscelinomys* lineages, with a derived form in OAM (25/18/25/3) caused by a pericentric inversion. The other branch is formed by three lineages and shows HME 18/2, 4/11/(16,17), and (13,22)/11 as signatures (Node C, Figure 4); this last is derived from HME (13,22)/5/11, possibly via translocation.

One branch comprises *Necromys* and *Thalpomys*, which exhibit loss of HME (13,22)/11, and another branch is composed of *Castoria* and *Thaptomys*, wherein multiple events have eliminated HME 18/2 and 4/11/(16,17). In the most recent divergent branch of *Akodon* and *Deltamys*, another ancestral trait (HME 18/25/3) is present as a derived character (HME 18/25) that arose via a fission event (Node D, Figure 4). We also identified a chromosomal signature (HME (9,10)/5) shared only by TNI and NLA, that reinforces the clade ((*Castoria* + *Thaptomys*) (*Necromys* + *Thalpomys*)) (Node E, Figure 4), and five specific traits for the *Akodon* genus (HME (16,17)/11/4/18/2/12/1, (13,22)/20/1, 23/2, 8/(13,22)/5/(16,17), and (9,10)/15).

Similar approaches have helped in the understanding about the evolution of other rodent species. As an example, da Silva et al. [46] plotted the chromosomal information (2n and FN) on a Cytb phylogeny of the *Neacomys* genus; the authors postulated that two increases and one reduction from the hypothetical ancestral 2n (56) occurred, which was in agreement with the molecular relationships retrieved by molecular data.

Although we did not have enough representative species in the comparative analysis to detect chromosomal signatures that support the monophyly of clade B, the chromosome-painting data of BBR (2n = 28, FN = 50) provide significant information. In particular, we found many syntenic associations that were not previously observed in the other taxa that have been investigated with HME probes (Figure 4; Supplementary Table S1), indicating that they are thus far exclusive to BBR, but could be shared with other representatives of clade B (HME (9,10)/7/25/3, 1/6/21, 11/23/18/4, (16,17)/8/1, (13,22)/20/2, 15/8 19/14/5, (9,10)/12, 7/(13,22)/5, (16,17)/18 and 26/24). The lack of more chromosomal signatures shared between clades A and B reinforces the separation of these two lineages, corroborating relationships recovered in the molecular analysis that reinforce the value of this approach in phylogenetic studies [5,27].

## 4. Materials and Methods

### 4.1. Samples

We studied the karyotypes of one male of *Oxymycterus amazonicus* (MZUFPAM 122) collected from Parauapebas municipality, Pará state, Brazil (05°21′54”S 49°07′24”W) and one male of *Blarinomys breviceps* (MN68882) collected from Santuário Serra da Concórdia, Valença municipality, Rio de Janeiro state, Brazil (22°22′18”S 43°47′23”W; Figure 1).

Samples were collected using Pitfall traps [66] and deposited at the zoological collections of Museu de Zoologia da Universidade Federal do Pará (UFPA), Belém, Pará, and the Museu Nacional da Universidade Federal do Rio de Janeiro (UFRJ), Rio de Janeiro, Rio de Janeiro. All institutions are in Brazil.

### 4.2. Cytogenetics

Chromosomal preparations were obtained from bone marrow [67]. Slides with chromosomal preparations were submitted to C-banding [68] and G-banding [69]. Twenty-four whole-chromosome probes from *Hylaeamys megacephalus* (HME) [23] were used for FISH experiments as described previously [23]; of these, three probes corresponded to two pairs of chromosomes each (HME (9,10), (13,22), and (16,17)).

### 4.3. Image Capture and Analysis 

Digital images of C- and G-banded karyotypes were obtained using an Olympus BX41 microscope with a CCD 1300QDS digital camera and analyzed using GenASIs software version 7.2.7.34276 from ASI (Applied Spectral Imaging). FISH images were obtained using a Nikon H550S microscope, a DS-Qi1Mc digital camera, and the Nis-Elements software. The karyotypes were organized according to established chromosomal morphology [70]. The final images were edited using Adobe Photoshop CS6.

## 5. Conclusions

We herein report new cytogenetic information for *Oxymycterus* and *Blarinomys* and distinct chromosomal evolutionary patterns for these genera. Our results show that while *Oxymycterus* exhibits a stable karyotype with few rearrangements (inversions and/or translocations), *Blarinomys* presents multiple Robertsonian translocations that reduced the 2n of this group. We propose that the speciation process of *Oxymycterus* was caused by vicariance events, and that chromosomal rearrangements did not play a crucial role in this process. We also suggest that the population structure of *Blarinomys breviceps* was established by the fragmentation of the Atlantic Forest and reinforced by chromosomal rearrangements. We used chromosomal signatures as an additional tool to elucidate the phylogenetic relationships among Akodontini clade A lineages, which reinforce the current low-supported arrangement of the branches retrieved by molecular data. Our comparative chromosome painting of Akodontini expands the analysis and may help improve our understanding of the evolutionary process and phylogenetic relationships in this extremely diverse group of rodents.

## Figures and Tables

**Table 1:** Akodontini tribe divisions and major clades recovered by the strict consensus tree from Maximum Parsimony analysis performed by D’Elía [27].

CLADE A				CLADE B	
Division	*Akodon*	*Oxymycterus*	*Bibimys*	*Blarinomys*	*Scapteromys*
**Genus**	*Akodon*	*Oxymycterus*	*Bibimys*	*Blarinomys*	*Scapteromys*
	*Deltamys*	*Juscelinomys*		*Brucepattersonius*	*Kunsia*
	*Necromys*			*Lenoxus*	
	*Thalpomys*				
	*Thaptomys*				
	* Castoria angustidens				

* Referred to as “*Akodon” serrensis* by D’Elía [23], but reviewed by Pardiñas et al. [28].

**Table 2:** Cytogenetic data available in the literature and obtained in the present study for *Oxymycterus* and *Blarinomys* genus. Abbreviations: 2n, diploid number; FN, fundamental autosomal number; B, B chromosomes. Numbers within parenthesis refer to the number of B chromosomes.

Species *	Karyotype	Reference
*Oxymycterus amazonicus*	2n = 54, FN = 64	Present study
*O. caparaoe*	2n = 54, FN = 64	[36]
*O. dasytrichus*	2n = 54, FN = 62	[37,38]
*O. delator*	2n = 54, FN = 62, 64	[36,39,40]
*O. nasutus*	2n = 54, FN = 64	[41]
*O. paramensis*	2n = 54, FN = 60, 64	[31,36]
*O. quaestor*	2n = 54, FN = 64	[36]
*O. rufus*	2n = 54, FN = 60, 64	[31,36]
*Oxymycterus* sp.	2n = 54, FN = 64	[29]
*Blarinomys breviceps*	2n = 52 (+2Bs), FN = 50; 2n = 52, FN = 50; 2n = 45 (+1B), FN = 50; 2n = 43 (+4Bs), FN = 50; 2n = 37 (+1B), FN = 50; 2n = 34, FN = 50; 2n = 31 (+2Bs), FN = 50; 2n = 28, FN = 50	[33,42]
*B. breviceps*	2n = 28, FN = 50	Present study

* We adopted the taxonomic classification proposed by Patton, Pardiñas, and D’Elía [20] when referring to species karyotypes.

**Table 3:** FISH signals detected for *Oxymycterus amazonicus* (OAM; 2n = 54, FN = 64) and *Blarinomys breviceps* (BBR; 2n = 28, FN = 50), as assessed based on hybridization with *Hylaeamys megacephalus* (HME) whole-chromosome probes [23].

HME	OAM	BBR
1	12, 13	2p, 4q
2	8	5q
3	1q dist.	1q dist.
4	9, 24	3q
5	2p prox., 3p, 23, 25	7q, 9q dist.
6	7q prox.	2q prox.
7	11q prox., 15	1p prox., 9p
8	10	4p prox., 6q
(9,10)	11q dist., 17	1p dist., 8p
11	2q dist., 16q prox.	3p dist.
12	18	8q
(13,22)	2p dist., 3q, 14q dist.	5p dist., 9q prox., 12
14	21q int., 26	7p proximal, 13
15	19	6p
(16,17)	4, 16q dist.	4p dist., 10p
18	1p prox., 5	3p prox., 10q
19	21q (prox. and dist.)	7p dist.
20	14q prox.	5p prox.
21	7q dist.	2q dist.
23	20	3p int.
24	22	11q
25	1p dist., 1q int.	1q prox.
26	6	11p
X	Xq	X

Short arm (p). Long arm (q). Proximal (prox). Interstitial (int). Distal (dist). Two segments (ts).

## Supplementary Materials

The following are available online at https://www.mdpi.com/1422-0067/21/7/2415/s1.
