# Supplementary material for: Chromosomal Signatures Corroborate the Phylogenetic Relationships within Akodontini (Rodentia, Sigmodontinae)
Source: Int J Mol Sci. 2020 Mar 31;21(7):2415. doi: 10.3390/ijms21072415 (PMC7177754; doi:10.3390/ijms21072415)
Supplement: Supplementary file 1 [file ijms-21-02415-s001.pdf]

Table 1. Chromosomal homology among species from Oryzomyini and Akodontini tribes, revealed with *Hylaeamys megacephalus* (HME) whole-chromosome probes [23].

	Oryzomyini											Akodontini				
**HME**	**CLA**	**OCA-PA**	**OCA-RJ**	**NSP-A**	**NSP-B**	**NSP-C**	**NSP-D**	**NPA**	**NSP-E**	**NAM**	**TNI**	**AMO**	**ASP**	**NLA**	**OAM**	**BBR**
**1**	2q, 20	13, 16, 29	13, 17, 28	6, 8	2p, 4q	6, 8	6, 8	6, 8	6, 8	6, 8	4, 8	1q dist., 4q	1q int., 2q dist.	5q dist., 7	12, 13	2p, 4q
**2**	10, 18, 19	4	1p, 4	2	3q	2	2	2	2	2	7, 12	1q int., 7q	1p dist., 2q int.	9q int., 13	8	5q
**3**	1q int., 3p	1	1q	3	2p	3	3	3	3	3	1 int. and dist.	2q	2p dist.	3q dist.	1q dist.	1q dist.
**4**	5, 13	2	2	1q dist.	1q dist.	1q dist.	1q dist.	1q dist.	1q dist.	1q dist.	13, 15	1p prox., 5p dist.	2q prox. and int., 3q int.	1q prox., 10q prox., 11q dist.	9, 24	3q
**5**	1p dist., 1q prox., 8	3q dist., 15, 27	3q dist., 15, 27	19, 22, 24, 26p	3p dist., 21, 22, 24	15q dist., 19, 24	15q dist., 19, 23	19, 26	9, 17	9, 28, 31	2 dist., 5 prox., 6 prox.	3q int., 6p int., 10	1p int. and prox., 3q int. (ts)	6q dist., 12q int., 14q dist.	2p prox., 3p, 23, 25	7q, 9q dist.
**6**	4q dist.	9, 25, 26	9, 16	5q prox., 18	5q prox., 8	5q prox., 18	5q prox., 18	5q prox., 18	5q prox., 19	5q prox., 18	3 prox. and int.	2p	2p int.	2q int.	7q prox.	2q prox.
**7**	3q int.	7	7	7, 9q int.	3p int., 5p	7, 9q int.	7, 9q int.	7, 9q int.	7, 10q int.	7, 10q int.	18	5q prox., 8q	1p int., 3q int.	1q int., 4q prox.	11q prox., 15	1p prox., 9p
**8**	4q prox., 7	5	5	12, 13	6, 15	12, 13	12, 13	12, 13	12, 16	12, 16	6 dist.	3p	3q prox.	1q int.	10	4p prox., 6q

**(9,10)**	2p dist., 3q dist.	3q prox., 12	3q prox., 12	9q (ts), 10	1p, 3p int. (ts)	9q (ts), 10	9q (ts), 10	9q (ts), 10	10q (ts), 14	10q (ts), 14	2 prox., 5 dist.	5q, 9p	1q int., 3q int.	1q dist., 6q prox.	11q dist., 17	1p dist., 8p
**11**	11q prox., 6	6q dist., 8 dist.	6q dist.; 8q dist.	20, 23p	11, 26	20, 22	20, 22q	14, 25p	24, 25	22, 23	9 dist., 10 prox.	1p int., 6q dist.	2q int., 3q dist.	10q int., 12q dist.	2q dist., 16q prox.	3p dist.
**12**	2p prox.	14	14	4q prox.	4q prox.	4q prox.	4q prox.	4q prox.	4q prox.	4q prox.	16	1q int.	2q int.	5q prox.	18	8q
**(13,22)**	1q (ts), 9	10q dist., 18q prox., 22	10q dist., 19q prox., 22	1q int., 26q, 27p	1q int., 19, 23	1q int., 21, 28p	1q int., 21, 28p	1q int., 20, 21	1q int., 21, 23	1q int., 19, 21	9 prox., 11 int. and dist., 21	3q prox., 4p dist., 6q prox.	1q int., 3q int. (ts)	4q dist., 12q prox., 14q prox.	2p dist., 3q, 14q dist.	5p dist., 9q prox., 12
**14**	1p int., 21	3q int., 30	3q int., 29	23q, 25p prox.	20, 25	25q, 26p prox.	24q, 25p prox.	16, 24p prox.	26, 28	24, 26	17 prox., 24	6p prox., 8p int.	1p int., 3q int.	2q int., 15q int.	21q int., 26	7p proximal, 13
**15**	12	11	11	15	9	17	17	15	15	15	19	9q	1q dist.	8q dist.	19	6p
**(16,17)**	1q prox., 11q dist.	19, 20	20, 24	4q dist., 16	4q dist., 10	4q dist., 16	4q dist., 16	4q dist., 25q	4q dist., 22	4q dist., 20	10 dist., 22	1p dist., 3q dist.	2p prox., 2q int., 3q int.	2q prox., 10q dist.	4, 16q dist.	4p dist., 10p
**18**	16	21	21	17	7	15q prox.,	15q prox.	17	27	25	1 prox., 23	1q prox., 5p prox.	2p int., 2q int., 3q int.	3q prox., 9q prox.	1p prox., 5	3p prox., 10q
**19**	1p int., 3q prox.	6q int., 28	6q int., 26	14, 25p dist.	18	14, 26p dist.	14, 25p dist.	24p dist., 24q	18	17	17 dist.	8p dist., 8q prox.	1p int. (ts)	15q prox. and dist.	21q (prox. and dist.)	7p dist.
**20**	1q dist.	10q prox.	10q prox.	1q prox.	1q prox.	1q prox.	1q prox.	1q prox.	1q prox.	1q prox.	11 prox.	4q prox.	1q prox. and int.	4q int.	14q prox.	5p prox.
**21**	4p, 4q int.	18q dist.	19q dist.	5q dist.	5q dist.	5q dist.	5q dist.	5q dist.	5q dist.	5q dist.	3 dist.	2p dist.	2p int.	2q dist.	7q dist.	2q dist.

**23**	15	6q prox., 23	6q prox., 23	21, 25q	14, 16	23, 26q	25q, 26q	23	13, 20	13, 29	20	7p	1p int.	8q prox.	20	3p int.
**24**	14	17	18	11	12	11	11	11	11	11	14	6p dist.	3q int. (ts)	9q dist.	22	11q
**25**	17	24	25	28	13	27	27	27	29	27	1 prox.	2p prox.	2p int., 3q int.	3q int., 11q prox.	1p dist., 1q int.	1q prox.
**26**	22	8q prox.	8q prox.	27q	17	28q	28q	22	30	30	25	11	4	16	6	11p
**X**	X	Xq	Xq	Xq	X	Xq	Xq	X	Xq	Xq	X	X (Xq)	X	X	Xq	X

Legend: Short arm (p). Long arm (q). Proximal (prox). Interstitial (int). Distal (dist). Two segments (ts). *Hylaeamys megacephalus* (HME), *Cerradomys langguthi* (CLA) [23], *O. catherinae*-Pará (OCA-PA), *O. catherinae*-Rio de Janeiro (OCA-RJ) [26], *Neacomys* sp. A (NSP-A), *Neacomys* sp. B (NSP-B) [8], *Neacomys* sp. C (NSP-C), *Neacomys* sp. D (NSP-D), *N. paracou* (NPA), *Neacomys* sp. E (NSP-E), and *N. amoenus* (NAM) [9], *Thaptomys nigrita* (TNI), *Akodon montensis* (AMO) [24], *Akodon* sp. (ASP), *Necromys lasiurus* (NLA) [25], *Oxymycterus amazonicus* (OAM), *Blarinomys breviceps* (BBR) (present study).
